# Elective pelvic nodal irradiation in the setting of ultrahypofractionated versus moderately hypofractionated and conventionally fractionated radiotherapy for prostate cancer: Outcomes from 3 prospective clinical trials

**DOI:** 10.1016/j.ctro.2024.100843

**Published:** 2024-08-16

**Authors:** Rachel M. Glicksman, Andrew Loblaw, Gerard Morton, Danny Vesprini, Ewa Szumacher, Hans T. Chung, William Chu, Stanley K. Liu, Chia-Lin Tseng, Melanie Davidson, Andrea Deabreu, Alexandre Mamedov, Liying Zhang, Patrick Cheung

**Affiliations:** a Department of Radiation Oncology, University of Toronto, Toronto, Canada; b Department of Radiation Oncology, Odette Cancer Centre, Sunnybrook Health Sciences Centre, Toronto, Canada; c Radiation Medicine Program, Princess Margaret Cancer Centre, University Health Network, Toronto, Canada; d Institute of Health Policy, Management and Evaluation, University of Toronto, Canada; e Department of Medical Physics, Kelowna General Hospital, BC Cancer, Kelowna, Canada; f Clinical Trials and Epidemiology Program, Odette Cancer Centre, Sunnybrook Health Sciences Centre, Toronto, Canada

**Keywords:** Ultrahypofractionation, Stereotactic body radiotherapy, Elective nodal irradiation, High-risk prostate cancer

## Abstract

**Background and purpose:**

Data is needed regarding the use of ultrahypofractionated radiotherapy (UHRT) in the context of prostate cancer elective nodal irradiation (ENI), and how this compares to conventionally fractionated radiotherapy (CFRT) ENI with CFRT or moderately hypofractionated radiotherapy (MHRT) to the prostate.

**Materials and methods:**

Between 2011–2019, 3 prospective clinical trials of unfavourable intermediate or high-risk prostate cancer receiving CFRT (78 Gy in 39 fractions to prostate; 46 Gy in 23 fractions to pelvis), MHRT (68 Gy in 25 fractions to prostate; 48 Gy to pelvis), or UHRT (35–40 Gy in 5 fractions to prostate +/- boost to 50 Gy to intraprostatic lesion; 25 Gy to pelvis) were conducted. Primary endpoints included biochemical failure (Phoenix definition), and acute and late toxicities (CTCAE v3.0/4.0).

**Results:**

Two-hundred-forty patients were enrolled: 90 (37.5 %) had CFRT, 90 (37.5 %) MHRT, and 60 (25 %) UHRT. Median follow-up time was 71.6 months (IQR 53.6–94.8). Cumulative incidence of biochemical failure (95 % CI) at 5-years was 11.7 % (3.5–19.8 %) for CFRT, 6.5 % (0.8–12.2 %) MHRT, and 1.8 % (0–5.2 %) UHRT, which was not significantly different between treatments (p = 0.38). Acute grade ≥ 2 genitourinary toxicity was significantly worse for UHRT versus CFRT and MHRT, but not for acute grade ≥ 3 genitourinary, or acute gastrointestinal toxicities. UHRT was not associated with worse late toxicities.

**Conclusion:**

ENI with UHRT resulted in similar oncologic outcomes to CFRT ENI with prostate CFRT/MHRT, with worse acute grade ≥ 2 GU toxicity but no differences in late toxicity. Randomized phase 3 trials of ENI using UHRT techniques are much anticipated.

## Highlights

• Three prospective trials evaluating elective nodal irradiation in the setting of ultrahypofractionated versus moderately hypofractionated and convetionally fractionated prostate radiotherapy were combined and analyzed.
• The cumulative incidence of biochemical failure was not significantly different between the cohorts.
• Acute grade ≥ 2 GU toxicity was significantly worse in patients treated with ultrahypofractionation, but not acute grade ≥ 3 GU toxicity.
• Elective nodal irradiation in the setting of prostate ultrahypofractionation was not associated with worse late toxicities.

## Introduction

Over the past two decades, there has been significant interest in hypofractionation in prostate cancer, owing to its unique radiobiology with low alpha–beta ratio compared to surrounding normal tissue [1], [2]. Initially, studies evaluated the role of moderately hypofractionated radiotherapy (MHRT) to the prostate, which demonstrated non-inferiority compared to conventionally fractionated radiotherapy (CFRT) [3], [4], [5], [6]. More recently, there has been interest in delivering even larger doses per fraction, and the efficacy of ultra-hypofractionated radiotherapy (UHRT) has been evaluated in two published phase 3 trials. The HYPO-RT-PC trial first demonstrated the non-inferiority of 7-fraction UHRT (using 3D conformal radiotherapy or intensity modulated radiotherapy) compared to 39-fraction CFRT [7]. More recently, the PACE-B trial demonstrated the non-inferiority of a 5-fraction UHRT regimen utilizing stereotactic body radiotherapy (SBRT) compared to either 20-fraction MHRT or 39-fraction CFRT [8].

However, the above studies have evaluated the role of hypofractionation when treating the prostate only, without including elective nodal irradiation (ENI). Although the role of ENI remains controversial, with two large phase 3 randomized trials, GETUG-1 and RTOG-9413 demonstrating equivalent event- or progression-free survival with ENI [9], [10], [11], [12]. It is notable that these studies were performed using relatively low radiotherapy doses to the prostate and shorter duration of androgen deprivation therapy (ADT) for patients with high-risk disease [13]. More recently, the randomized phase 3 POP-RT trial demonstrated significant oncologic improvement of biochemical progression-free survival and distant metastasis-free survival with the addition of ENI, with patients treated with MHRT [14]. Importantly, POP-RT included a well-selected patient population of high-risk disease patients the majority who had undergone prostate specific membrane antigen (PSMA) positron emission tomography (PET) staging [14].

Few studies have assessed the role of ENI treated with UHRT. A *meta*-analysis of 417 patients across 7 publications was performed with a median follow up of 3 years, and focused on toxicity outcomes [15]. The SPORT trial, a feasibility trial of 30 patients randomized patients between prostate stereotactic body radiotherapy (SBRT) with or without ENI, and found increased rates of late grade ≥ 2 GI and GU toxicity in the group receiving ENI with median follow up at 3 years [16].

With the increasing use of UHRT for management of the prostate [17], more data is needed regarding the use of ENI in the setting of UHRT, and how this treatment compares to CFRT ENI in the context of CFRT or MHRT to the prostate. Herein, we present results from 3 prospective clinical trials that included CFRT, MHRT, and UHRT to the prostate and ENI for unfavourable prostate cancer.

## Materials and methods

All three prospective phase 2 trials were approved by the Sunnybrook Research Ethics Board and registered on clinicaltrials.gov (NCT04239599 for pHART2; NCT01953055 for SATURN, and NCT02911636 for 5STAR). All studies used the 2009 RTOG atlas for pelvic nodal clinical target volumes (CTV) [18] with a 6 mm planning target volume (PTV).

### Patient selection and treatment details

From 2011 to 2019, 186 patients (of which 180 were eligible for analysis: 90 randomized to MHRT and 90 randomized to CFRT) with high-risk prostate cancer were enrolled in pHART2. Study details were previously published [19]. Briefly, patients were randomized to either MHRT using a simultaneous integrated boost or CFRT. Patients randomized to MHRT received 68 Gy in 25 fractions to prostate and 48 Gy to pelvis, with a 4 mm isotropic margin around the primary CTV (prostate and proximal seminal vesicles (SVs) or the entire SV’s for clinical stage T3b disease). All MHRT patients underwent transperineal implantation of 3 gold prostate fiducial markers prior to computed tomography (CT) simulation. No rectal spacers were used. Patients randomized to CFRT received 46 Gy in 23 fractions with a sequential boost to the prostate of 32 Gy in 16 fractions, with a 10 mm margin (except 7 mm posteriorly) around the primary CTV and primary CTV boost. Daily cone beam CT (CBCT) was performed for image guidance in both arms. In the MH arm, the implanted fiducial markers were used for matching, while soft tissue matching was used in the CF arm (which is why the PTV margins were different between the 2 arms). Patients received 1.5–3 years of androgen deprivation therapy (ADT).

Between 2013–2014, 30 patients with high-risk prostate cancer were enrolled in SATURN. Study details have been reported [20]. CTV1 encompassed pelvic lymph nodes and SVs, with a 6 mm margin for planning target volume-1 (PTV1). CTV2 encompassed the prostate, with a 3 mm margin for PTV2. Planning doses were 25 Gy to CTV1, 23.25 Gy to PTV1, 40 Gy to CTV2, and 33.25 Gy to PTV2, in 5 weekly fractions. All patients underwent transperineal implantation of 3 gold fiducial markers prior to CT simulation, which was performed using a custom vacuum lock bag for immobilization. No rectal spacers were used. Daily CBCT was performed for image guidance. The implanted fiducial markers were used for matching. Patients received 12–18 months of ADT.

From 2016 to 2017, 30 patients with unfavourable-intermediate or high-risk prostate cancer were enrolled in 5STAR. Study details have been published [21]. Briefly, patients were treated with 25 Gy to the pelvic lymph nodes and SVs, and 35 Gy to the prostate with a simultaneous integrated boost up to 50 Gy to a magnetic resonance (MR)-detected intraprostatic nodule, delivered in 5 weekly fractions. CTV1, CTV2, and PTV1 were created the same as in the SATURN study. PTV2 was created using a 2 mm expansion (except 2.5 mm superiorly-inferiorly) on CTV2, enabled through the use of a prostate-endorectal immobilization system [22]. All patients underwent transperineal implantation of 3 gold fiducial markers prior to CT simulation and fused MR. No rectal spacers were used. Daily CBCT was performed for image guidance. The implanted fiducial markers were used for matching. Urethrogram was performed at the time of planning to identify the urethra as a dose-limiting structure. The dominant intraprostatic lesion (DIL) was contoured on the fused multiparametric magnetic resonance imaging and boosted up to 50 Gy if the organs-at-risk dose limits were not exceeded. Patients received 6–18 months of ADT.

### Study endpoints and follow-up

Patients were assessed at baseline, weekly during radiotherapy, every 3–6 months for 5 years, and then once a year or more frequently as clinically indicated. Prostate specific antigen (PSA) and testosterone were measured at baseline and each follow-up visit following radiotherapy. Imaging was not routinely performed, but may have been at the discretion of the physician at the time of biochemical failure, either with conventional imaging (CT and/or bone scan and/or MR) or prostate-specific membrane antigen (PSMA) positron emission tomography (PET)-CT. Salvage therapies including salvage ADT was performed at physician and patient discretion, without a study-mandated pre-determined threshold. Biochemical failure was defined as per the Phoenix definition (nadir plus 2.0 ng/mL). Distant metastasis was defined as the presence of extra-prostatic disease on any form of imaging. Castrate-resistant prostate cancer (CRPC) was defined as rising PSA or development of new metastases in the setting of castrate testosterone levels. Toxicities were collected using Common Terminology Criteria for Adverse Events (CTCAE) versions 3.0 (pHART2 and SATURN) and 4.0 (5STAR).

### Statistical analysis

Demographic and tumor characteristics were summarized using median and interquartile range (IQR) for continuous variables and proportions for categorical variables. Estimates of risk of lymph node involvement (LNI) were calculated according to the Roach formula [23]. Cumulative incidence of biochemical failure, CRPC, and acute and late toxicities were estimated using Nelson-Aalen estimates. Metastasis-free survival, prostate cancer-specific survival (PCSS), and overall survival (OS) were estimated using Kaplan-Meier method. Univariate and multivariable Cox proportional hazards model on predictive factors were performed for oncologic outcomes and toxicity, with variables assessed including age, treatment type (CFRT vs MHRT vs UHRT), modality type (IMRT vs SBRT), risk group (unfavorable intermediate vs high risk), use of ADT (yes vs no), and duration of ADT (logarithm transformed). P<0.05 was considered statistically significant, all analyses were conducted using Statistical Analysis Software (v9.4, Cary, NC) and R package (v4.3.0).

## Results

240 patients were included: 90 patients received CFRT, 90 received MHRT, and 60 received UHRT. Baseline characteristics are outlined in Table 1. The groups differed significantly in risk group categories, as the UHRT trials allowed unfavorable intermediate risk patients (n = 13), whereas only patients with high-risk disease received CFRT and MHRT (p < 0.0001). Median estimates of risk of LNI also significantly differed, with patients receiving CFRT and MHRT having significantly higher estimated risk of LNI (33.1 % and 32.9 % risks, respectively) compared to patients receiving UHRT (26.9 % risk) (p = 0.0006). However, within the UHRT group, patients with high-risk disease had significantly higher median risk of LNI (28.1 %) compared to the unfavourable intermediate risk disease patients (18.8 %) (p < 0.0001). Groups also differed in duration of ADT, with patients receiving UHRT having significantly shorter durations of ADT (p < 0.0001). Median follow up was 71.6 months (IQR 53.6–94.8) for the entire cohort and was 65.6 months (IQR 49–87.9) for CFRT, 72.5 months (IQR 46.7–95.7) for MHRT, and 77.2 months (IQR 66.6–98.4) for UHRT. Results are reported as aggregates across all trials.

**Table 1:** Baseline patient and treatment characteristics.

Variables	CFRT (n = 90)	MHRT (n = 90)	UHRT (n = 60)	p-value
Age (median, IQR)	77 (71–80)	75 (70–79)	74 (68–79)	0.1
Risk group (n, %) UIR High risk	0 90 (100 %)	0 90 (100 %)	13 (21.7 %) 47 (78.3 %)	<0.0001
Grade group (n, %) 1 2 3 4 5 Unknown	0 14 (15.6 %) 10 (11.1 %) 33 (36.7 %) 29 (32.2 %) 4 (4.4 %)	1 (1.1 %) 5 (5.6 %) 8 (8.9 %) 39 (43.3 %) 32 (35.6 %) 5 (5.6 %)	1 (1.7 %) 10 (16.7 %) 15 (25 %) 18 (30 %) 16 (26.7 %) 0 (0 %)	0.03
Clinical stage (n, %) T1c T2a T2b T2c T3a T3b Unknown	28 (31.1 %) 27 (30 %) 14 (15.6 %) 8 (8.9 %) 8 (8.9 %) 1 (1.1 %) 4 (4.4 %)	30 (33.3 %) 23 (25.6 %) 12 (13.3 %) 7 (7.8 %) 12 (13.3 %) 1 (1.1 %) 5 (5.6 %)	10 (16.7 %) 17 (28.3 %) 10 (16.7 %) 11 (18.3 %) 8 (13.3 %) 3 (5 %) 1 (1.7 %)	0.3
Baseline PSA (median, IQR)	15.7 (7.1–26.5)	13.5 (7.5–23.6)	11.8 (6.9–19.9)	0.3
Baseline PSA categories (n, %) <10 10–20 >20 Unknown	31 (34.4 %) 21 (23.3 %) 35 (38.9 %) 3 (3.3 %)	30 (33.3 %) 27 (30 %) 29 (32.2 %) 4 (4.4 %)	22 (36.7 %) 23 (38.3 %) 15 (25 %) 0 (0 %)	0.3
Risk of lymph node involvement (median, IQR)	33.1 (10.4–73.7)	32.9 (17.9–80.0)	26.9 (21.7–33.0)	0.0006
Duration of ADT (median, IQR)	22.2 (15–29.5)	22 (15–30.1)	12 (6.2–15.9)	<0.0001
Duration of ADT categories (n, %) 0 months ≤12 months >12–24 months ≥24 months	5 (5.6 %) 17 (18.9 %) 29 (32.2 %) 39 (43.3 %)	3 (3.3 %) 15 (16.7 %) 35 (38.9 %) 37 (41.1 %)	4 (6.6 %) 24 (40 %) 31 (51.7 %) 1 (1.7 %)	<0.0001

*CFRT: conventionally fractionated radiotherapy; MHRT: moderately hypofractionated radiotherapy; UHRT: ultrahypofractionated radiotherapy; IQR: interquartile range; UIR: unfavorable intermediate risk; PSA: prostate specific antigen; ADT: androgen deprivation therapy.

### Oncologic outcomes

**Fig. 1:**
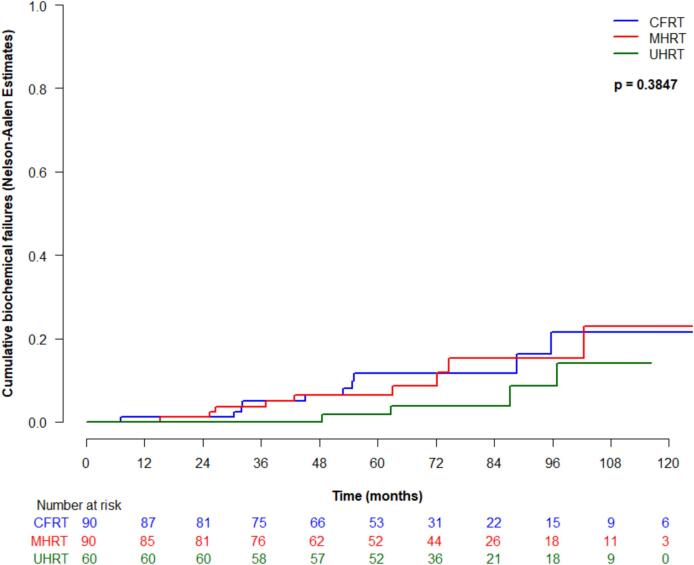
Oncologic outcomes by treatment type: (a) cumulative incidence of biochemical failure; (b) Kaplan-Meier of metastasis-free survival; (c) cumulative incidence of castrate-resistant prostate cancer; (d) Kaplan-Meier of prostate cancer specific survival; and (e) Kaplan-Meier of overall survival.

Cumulative incidence of biochemical failure (95 % CI) at 5-years was 11.7 % (3.5–19.8 %) for CFRT, 6.5 % (0.8–12.2 %) for MHRT, and 1.8 % (0–5.2 %) for UHRT, which was not significantly different between treatment types (p = 0.38) (Fig. 1a). Excluding the unfavourable intermediate risk patients from the UHRT group, the cumulative incidence of biochemical failure at 5 years for UHRT for high-risk patients exclusively was 2.3 % (95 % CI 0–4.6 %). There were no significant predictive factors associated with biochemical failure on univariate analysis. Metastasis-free survival was not significantly different between treatment type (p = 0.68), and at 5-years was 98.7 % (95 % CI 96.1–100 %) for CFRT, 98.7 % (96.2–100 %) for MHRT, and 98.2 % (94.9–100 %) for UHRT. There were no significant predictive factors associated with metastasis-free survival on univariate analysis.

Cumulative incidence of CRPC at 5-years (95 % CI) was 1.2 % (0.1–5.9 %) for CFRT, 0 % for MHRT, and 0 % for UHRT (p = 0.54), with no significant predictive factors on univariate analysis. PCSS was not significantly different between treatment type (p = 0.35), and at 5-years was 98.7 % (95 % CI 96.2–100 %) for CFRT, 100 % for MHRT and 100 % for UHRT, with no significant predictive factors on univariate analysis. At 5-years, overall survival (95 % CI) was 90.8 % (84.5–97.5 %) for CFRT, 91.3 % (85.4–97.7 %) for MHRT, and 93.3 % (87.2–99.9 %) for UHRT, and was not significantly different between the groups (p = 0.74). On univariate analysis, the use of ADT was significantly associated with survival, with patients receiving ADT having a lower probability of death compared to patients who did not receive ADT (HR 0.24, 95 % CI 7.7–74.3, p = 0.01).

### Acute toxicity

**Fig. 2:**
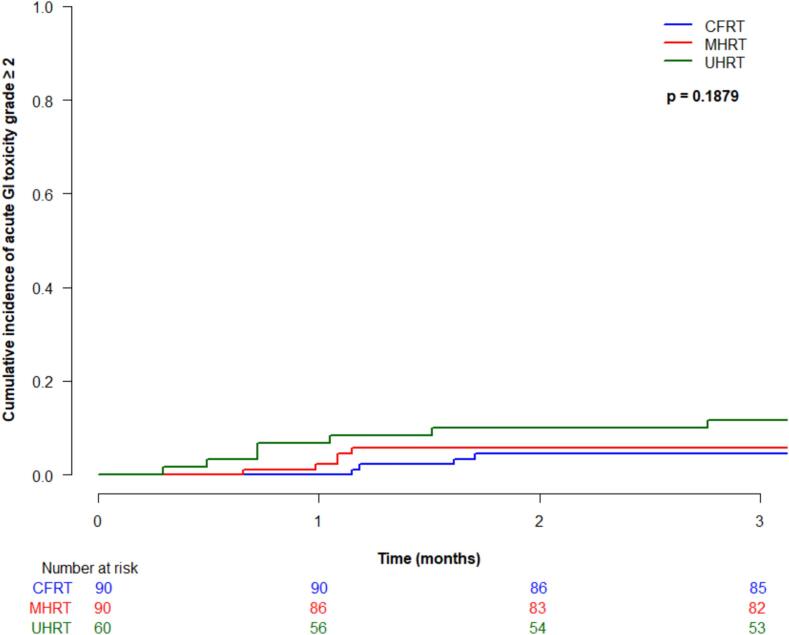

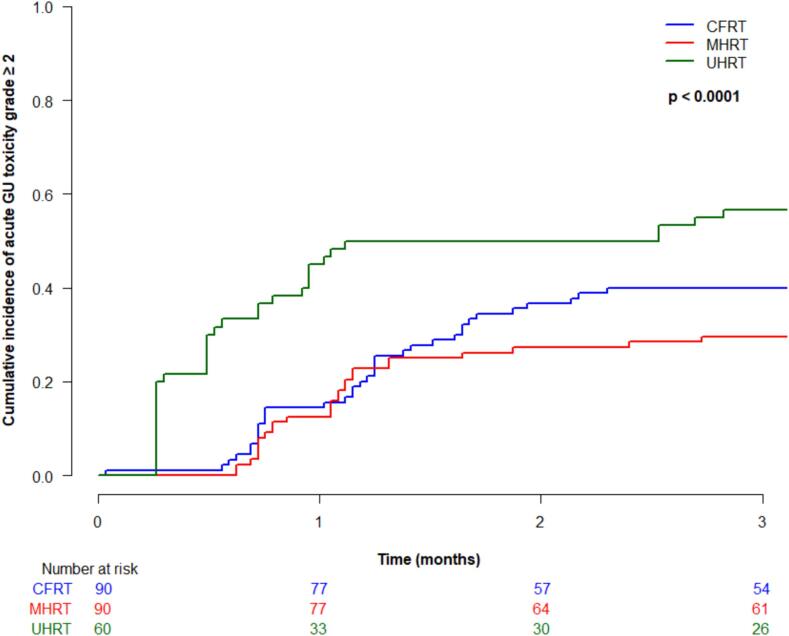

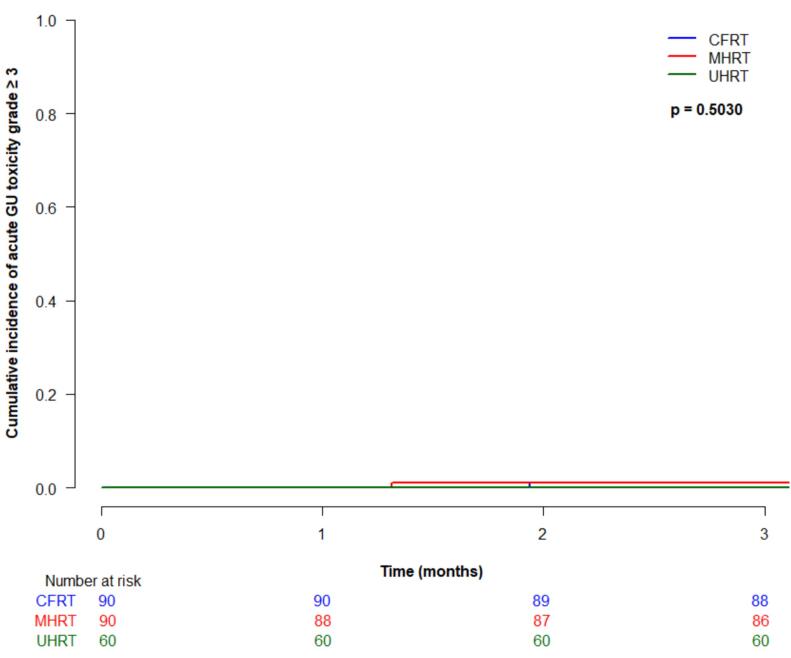
Acute toxicity by treatment type: (a) grade ≥ 2 gastrointestinal; (b) grade ≥ 2 genitourinary; (c) grade ≥ 3 genitourinary.

Cumulative incidence (95 % CI) of acute grade ≥ 2 gastrointestinal (GI) toxicity at 3-months was 4.4 % (1.4–10.2 %) for CFRT, 5.7 % (2.1–11.9 %) for MHRT, and 11.7 % (5.1–21.2 %) for UHRT, which was not significantly different between treatment type (p = 0.19) (Fig. 2a). There were no acute grade ≥ 3 GI toxicities in any patients. Acute grade ≥ 2 genitourinary (GU) toxicity at 3 months was 40 % (95 % CI 29.8–50 %) for CFRT, 30 % (20.4–39.3 %) for MHRT, and 56.7 % (43–68.2 %) for UHRT, which was statistically significantly different between groups (p < 0.0001) (Fig. 2b). On multivariable analysis, treatment type was significantly associated with acute grade ≥ 2 GU toxicity (UHRT vs CFRT HR 2.1, 95 % CI 1.3–3.2, p = 0.0009, and UHRT vs MHRT HR 2.9, 95 % CI 1.8–4.7, p < 0.0001). Acute grade ≥ 3 GU toxicity was rare, with cumulative incidences (95 % CI) at 3 months of 1.1 % (0.1–5.4 %) for CFRT, 1.1 % (0.1–5.6 %) for MHRT, and 0 % for UHRT, not significantly different between the groups (p = 0.50).

### Late toxicity

**Fig. 3:**
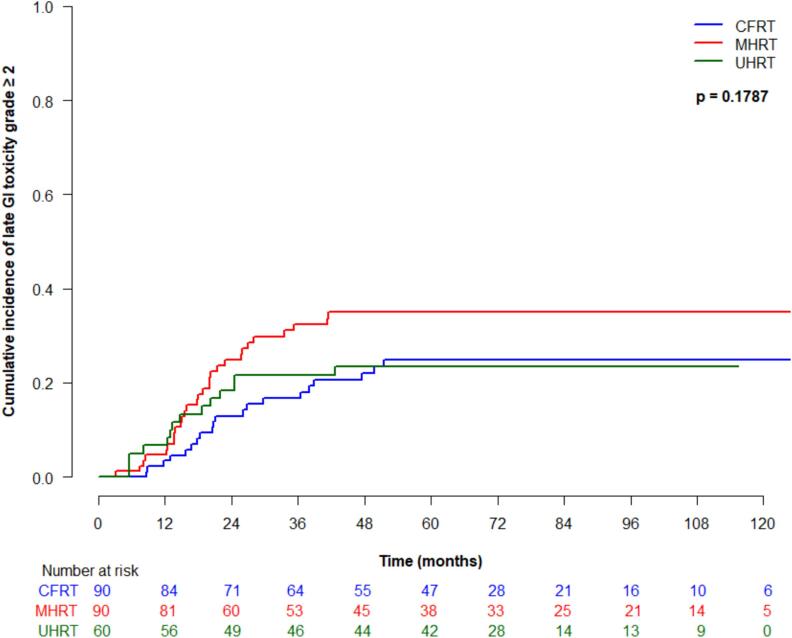

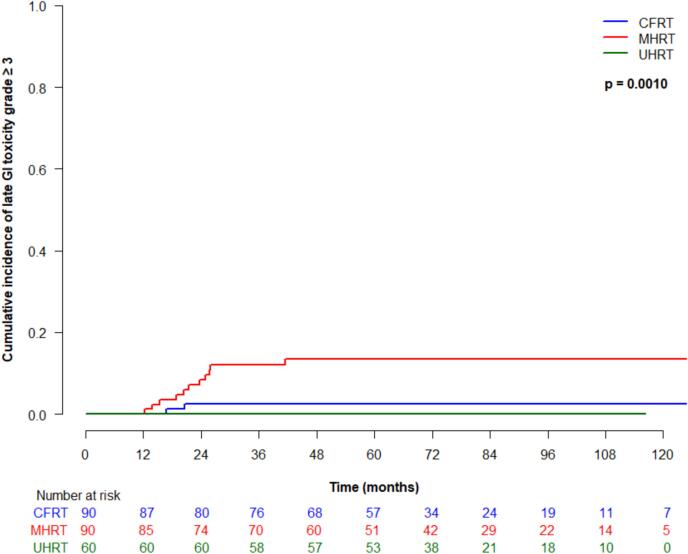

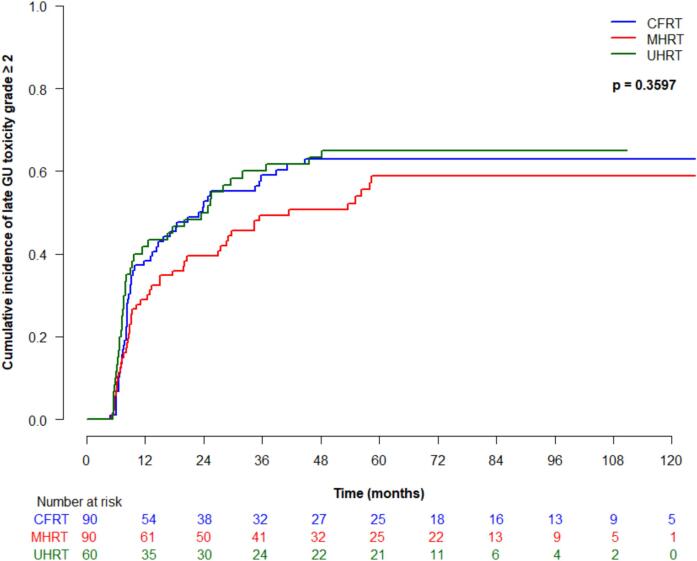

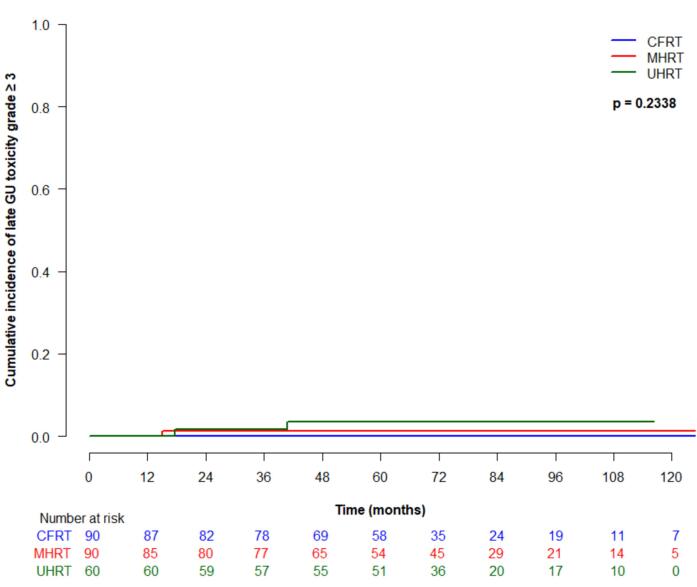
Late toxicity by treatment type: (a) grade ≥ 2 gastrointestinal; (b) grade ≥ 3 gastrointestinal; (c) grade ≥ 2 genitourinary; (d) grade ≥ 3 genitourinary.

Cumulative incidence (95 % CI) of late grade ≥ 2 GI toxicity at 5 years was 25 % (16–35 %) for CFRT, 35 % (24.9–45.4 %) for MHRT, and 23.4 % (95 % CI 13.6–34.8 %) for UHRT, which was not significantly different between treatment types (p = 0.18) (Fig. 3a). On univariate analysis, older age was significantly associated with higher risk of developing late grade ≥ 2 GI toxicity (HR 1.1, 95 % CI 1.0–1.1, p = 0.02). Late grade ≥ 3 GI toxicity at 5 years was 2.4 % (95 % CI 0.5–7.6 %) for CFRT, 13.4 (7.1–21.7 %) for MHRT and 0 % for UHRT, which was significantly different between groups (p = 0.001) (Fig. 3b). On univariate analysis, treatment type was significantly associated with late grade ≥ 3 GI toxicity (p = 0.02), and significantly differed for MHRT vs CFRT (HR 4.8, 95 % CI 1.1–20.4, p = 0.03). Cumulative incidence (95 % CI) of late grade ≥ 2 GU toxicity at 5 years was 63 % (51.5–72.5 %) for CFRT, 58.8 % (46.8–69 %) for MHRT, and 65 % (51.3–75.7 %) for UHRT, and was not significantly different between treatment groups (p = 0.36) (Fig. 3c). Late grade ≥ 3 GU toxicity (95 % CI) at 5 years for CFRT, MHRT, and UHRT was 0 %, 1.2 % (0.1–5.7 %), and 3.4 % (2.4–10.5 %), respectively, and was not significantly different amongst treatment types (Fig. 3d).

## Discussion

This analysis shows that with a median follow-up of approximately 6 years, there were no statistically significant differences in oncologic outcomes between the various fractionation types. This mirrors the evidence to date examining hypofractionation in the setting of prostate-only radiotherapy. Interestingly, the cumulative incidences of biochemical failure were numerically different among the groups (<2% for UHRT, >10 % for CFRT), although not statistically significant, perhaps owing to the under-powered nature of this analysis, or differences in baseline patient characteristics between the studies including a higher estimated risk of LNI in patients receiving CFRT and MHRT compared to UHRT which may have driven these oncologic results, although the high-risk disease patients who received UHRT had similar estimates of risk of LNI compared to those receiving CFRT and MHRT. Interestingly, similar findings were seen in PACE-B with prostate-only radiotherapy, where the 5-year biochemical failure event-free survival rate was numerically (but not statistically) improved for UHRT (SBRT) compared to the standard arm.

In terms of toxicity, we found that UHRT was associated with significantly worse acute grade ≥ 2 GU toxicity. Fortunately, there was no worse acute grade ≥ 3 GU toxicity, and the toxicity seems to have been transient, as there was no worse late GU toxicities in the UHRT arm compared to CFRT or MHRT. Grade 2 GU toxicities may be influenced by prescribing patterns for medications such as alpha adrenergic receptor antagonists and beta-3 adrenergic receptor agonists. Furthermore, half of the patients in the UHRT group received a DIL boost which could be associated with higher incidence of GU toxicity. Although our prior analysis of the two UHRT trials included in this analysis did not demonstrate statistically worse GU toxicity with a DIL boost, this may be owing to underpower with small sample sizes, as the numeric differences in acute grade ≥ 2 toxicity were large (66.6 % with DIL boost versus 43.3 % without) [21) Further, an interim analysis from the PRIME trial (NCT03561961) of UHRT versus MHRT to the prostate and pelvis did not demonstrate significant differences in acute grade ≥ 2 GU toxicity [24]. UHRT did not have any significantly worse late toxicities compared to the other treatment types. Interestingly, the 1-year urinary flare leading to worse GU toxicities in PACE-B was not observed in the present study [25]. The only significant difference in late toxicities was the worse grade ≥ 3 GI toxicity in MHRT, despite use of a tighter PTV margin, which we initially demonstrated in our analysis of the pHART2 study [19], but there were no differences between UHRT and CFRT.

Further study of UHRT for prostate and ENI treatment is needed. To our knowledge, five phase 3 randomized controlled trials investigating UHRT are ongoing: four trials are comparing different fractionation schemes, and one is assessing the role of ENI compared to prostate only radiotherapy. First, the PRIME trial (NCT03561961) is a non-inferiority multi-centre study of 464 patients with high risk localized or node positive PCa randomized to MHRT (50 Gy to the pelvis and 66–68 Gy in 25 fractions to the prostate, delivered daily) or UHRT (25 Gy to the pelvis and 35–36.25 Gy in 5 fractions to the prostate, delivered daily or alternate days) with a primary endpoint of 4-year biochemical failure free survival [26] Second, the ‘High Five Trial’ − NRG GU013 (NCT05946213) is a randomized non-inferiority multi-centre trial of 1209 patients with high risk localized or node positive PCa randomized to CFRT or MHRT (20–45 fractions over 4–9 weeks) or UHRT (5 fractions delivered on alternate days) with a primary endpoint of metastasis-free survival.

Third, ASCENDE-SBRT − CCTG PR.24 is a multi-centre study of 710 patients with high risk localized PCa investigating high-dose rate or low-dose rate brachytherapy boost plus external beam radiotherapy to the prostate and pelvis (46 Gy in 23 fractions) versus SBRT to the prostate and pelvis (25 Gy to the pelvis and 40 Gy to the prostate). Fourth, PCS-XI (NCT05820633) is a non-inferiority multi-centre randomized study of 500 patients with prostate cancer needing pelvic RT investigating CFRT or UHRT (25 Gy in 5 fractions) to the prostate and pelvis in combination with a HDR brachytherapy boost with a primary endpoint of toxicity and quality of life.

Fifth, PACE-NODES (NCT05613023) is a multi-centre trial aiming to accrue 536 patients with high risk localized PCa investigating the role of pelvic RT in the context of UHRT. In PACE-NODES, patients are randomized to either receive prostate only SBRT (36.25 Gy in 5 fractions to the prostate and SVs with a dose of 40 Gy to the prostate CTV, delivered on alternate days) or prostate and pelvic SBRT (25 Gy to the pelvis and 36.25 Gy in 5 fractions to the prostate and SVs with a dose of 40 Gy to the prostate CTV, delivered on alternate days) with a primary endpoint of time to biochemical or clinical failure at a minimum of 3.5 years of follow-up.

This analysis must be interpreted in the context of the limitations of the study. First, this cohort was developed from 3 separate prospective trials with different enrolment criteria, with the allowance of unfavourable intermediate risk patients into the UHRT trials but not CFRT or MHRT trial. Patients on the UHRT trials also received a shorter duration of ADT compared to the other patients, so the efficacy results should be interpreted with that in mind. Second, the studies took place over different time periods, although the UHRT trials took place over the timespan of pHART2 RCT. This also contributed to 5STAR using an updated CTCAE scale for toxicity grading compared to pHART2 RCT and SATURN. Third, SATURN and 5STAR were single-arm studies without a comparator arm of MHRT or CFRT radiotherapy, and pHART2 RCT did not include an UHRT arm. Fourth, SATURN and 5STAR used two different prostate doses, and although the results are presented as aggregate, there may be differences related to prostate dose. Overall, this data should be viewed as hypothesis generating. Results from phase 3 randomized studies of UHRT versus MHRT or CFRT are needed to determine the efficacy of an UHRT approach before it is routinely adopted into clinical practice.

In summary, ENI using UHRT resulted in similar oncologic outcomes to CFRT and MHRT, with worse acute grade ≥ 2 GU toxicity but no differences in late toxicity. Randomized phase 3 trials of ENI using UHRT techniques are ongoing and much anticipated.

## CRediT authorship contribution statement

**Rachel M. Glicksman:** Writing – original draft, Writing – review & editing, Methodology. **Andrew Loblaw:** Writing – original draft, Writing – review & editing, Methodology, Supervision, Funding acquisition. **Gerard Morton:** Writing – review & editing. **Danny Vesprini:** Writing – review & editing. **Ewa Szumacher:** Writing – review & editing. **Hans T. Chung:** Writing – review & editing. **William Chu:** Writing – review & editing. **Stanley K. Liu:** Writing – review & editing. **Chia-Lin Tseng:** Writing – review & editing. **Melanie Davidson:** Writing – review & editing. **Andrea Deabreu:** Investigation, Data curation. **Alexandre Mamedov:** Data curation. **Liying Zhang:** Software, Formal analysis. **Patrick Cheung:** Writing – original draft, Writing – review & editing, Methodology, Supervision, Funding acquisition, Project administration.

## Declaration of competing interest

The authors declare the following financial interests/personal relationships which may be considered as potential competing interests: RMG reports honoraria from Bayer, Tolmar, Knight, and TerSera; Research funding from Astellas and TerSera. CLT reports travel accommodations/expenses & honoraria for past educational seminars by Elekta; belongs to the Elekta MR-Linac Consortium; advisor/consultant with Sanofi and AbbVie. AL reports honoraria/travel from AbbVie, Astellas, Bayer, Janssen, Knight, TerSera; Grant/research support from TerSera and Tolmar; Advisory boards/consulting for Astellas, Bayer, Janssen, Sanofi, Tolmar and TerSera; Patents/licenses for endorectal immobilization device (GU-Lok); Employment: spouse is national sales director, Sanofi Canada. PC reports honoraria from TerSera and AstraZeneca. All other authors report no disclosures.
